# Fully automated segmentation of callus by micro-CT compared to biomechanics

**DOI:** 10.1186/s13018-017-0609-9

**Published:** 2017-07-11

**Authors:** Oliver Bissinger, Carolin Götz, Klaus-Dietrich Wolff, Alexander Hapfelmeier, Peter Michael Prodinger, Thomas Tischer

**Affiliations:** 1 Department of Oral and Maxillofacial Surgery, Klinikum rechts der Isar der Technischen Universität München, Ismaninger Str. 22, 81675 Munich, Germany; 2 Institute of Medical Statistics and Epidemiology, Klinikum rechts der Isar der Technischen Universität München, Ismaninger Str. 22, 81675 Munich, Germany; 3 Department of Orthopaedics and Orthopaedic Sports Medicine, Klinikum rechts der Isar der Technischen Universität München, Ismaninger Str. 22, 81675 Munich, Germany; 4 Department of Orthopaedic Surgery, University of Rostock, Doberanerstr. 142, 18057 Rostock, Germany

**Keywords:** Fracture healing, Micro-CT (μCT), Biomechanics, Fully automated segmentation, Comminuted fracture, Multi-fragmented fracture, Trabecular bone, 3D structural parameters

## Abstract

**Background:**

A high percentage of closed femur fractures have slight comminution. Using micro-CT (μCT), multiple fragment segmentation is much more difficult than segmentation of unfractured or osteotomied bone. Manual or semi-automated segmentation has been performed to date. However, such segmentation is extremely laborious, time-consuming and error-prone. Our aim was to therefore apply a fully automated segmentation algorithm to determine μCT parameters and examine their association with biomechanics.

**Methods:**

The femura of 64 rats taken after randomised inhibitory or neutral medication, in terms of the effect on fracture healing, and controls were closed fractured after a Kirschner wire was inserted. After 21 days, μCT and biomechanical parameters were determined by a fully automated method and correlated (Pearson’s correlation).

**Results:**

The fully automated segmentation algorithm automatically detected bone and simultaneously separated cortical bone from callus without requiring ROI selection for each single bony structure. We found an association of structural callus parameters obtained by μCT to the biomechanical properties. However, results were only explicable by additionally considering the callus location.

**Conclusions:**

A large number of slightly comminuted fractures in combination with therapies that influence the callus qualitatively and/or quantitatively considerably affects the association between μCT and biomechanics. In the future, contrast-enhanced μCT imaging of the callus cartilage might provide more information to improve the non-destructive and non-invasive prediction of callus mechanical properties. As studies evaluating such important drugs increase, fully automated segmentation appears to be clinically important.

## Background

Osteoporosis is frequently regarded as a pathology of cancellous bone, which is the reason why biomechanics and trabecular structures within the bone are of high socioeconomic interest. Human bone biopsies were originally collected from different donor sites (e.g. femoral head, vertebral bodies and iliac crest) to develop three-dimensional (3D) methods for the direct quantification of the actual structure type of cancellous bone. Therefore, no assumptions regarding the structure type (e.g. ‘plate-like’ or ‘rod-like’) are needed [1]. These micro-CT (μCT) measures were then correlated to the biomechanical parameters of the biopsies to determine their predictive value for the mechanical properties of bone samples from healthy individuals [2]. As several significant relationships were determined, various fracture healing studies were performed. For example, murine femur fracture callus mechanical properties were predicted by several μCT-derived basic morphometric indices of callus structure (particularly TMD and BV) [3–5].

Parameters of trabecular morphometry, such as trabecular thickness (Tb. Th.), structure model index (SMI) and degree of anisotropy (DA), are rarely reported as a group. Such omissions may occur because they show lower statistical significance combined with a need for more demanding interpretation. However, these measures should be routinely described because of their potential in future preclinical and clinical studies as surrogate measures of callus mechanical properties [6].

Eighty-five percent of the skeleton consists of cortical bone providing the majority of mechanical strength and stiffness [7]. However, following a fracture, the situation changes. The callus (peri- and endosteal), which mainly consists of trabecular bone, has to maintain relevant components of stability [8]. The size and shape of the callus can vary extensively; therefore, segmentation of this type of tissue is much more difficult than that of unfractured bone [9].

**Fig. 1:**
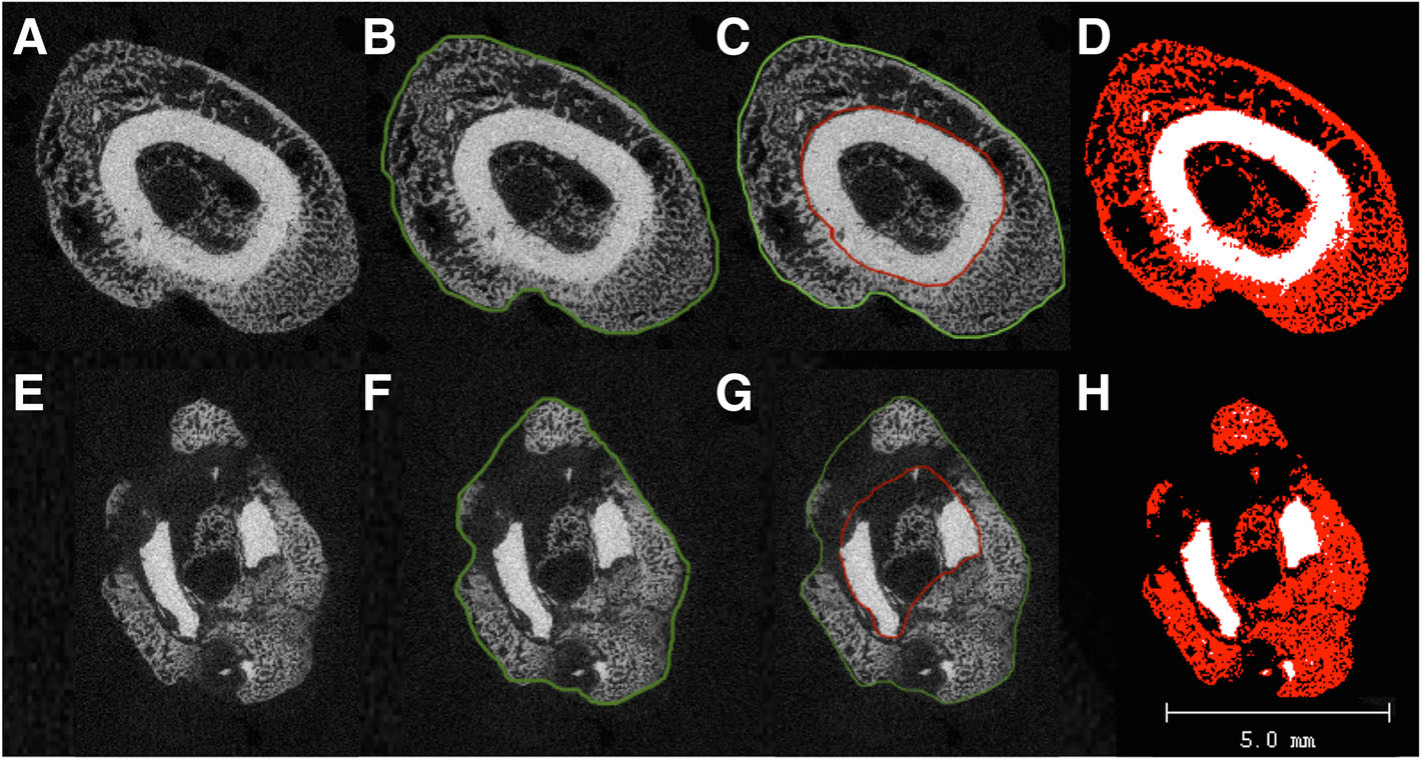
2D axial μCT grey-value image of the original cortical bone, callus and air/bone marrow (**a**, **e**), conventionally contoured images according to the method described by Namayn et al. (**b**) and Morgan et al. (**c**) and a corresponding fully automated segmented image (**d**, callus = *red*, original bone = *white*, marrow/air = *black*). A single contour adjacent to the callus distorted the evaluation of the volume-dependent callus parameters (e.g. BV/TV and BMD) because TV enclosed (in addition to the callus) all structures within the contour, such as the cortical bone and air/marrow. Therefore, the adjacent contouring is not necessary (**b**, **f**). Contouring of both the outer callus (*green line*) and outer cortical bone (*red line*) enables all callus parameters to be determined (*between the lines*) in an area not corresponding to the fracture gap (**c**). The latter was not possible within the fracture gap, especially if slight comminution was present (**g**). The enclosed air and unmineralised tissue would contribute to considerable underestimation of the volume-dependent parameters, and no measurement of the endosteal callus would be possible. Fully automated segmentation required no adjacent contouring of the ROI and enabled, even within the fracture gap, the separation of all islands of callus and cortical bone as well as determination of the non-volume-dependent parameters (**h**; for ROI, see Fig. 3). *Upper line*: the same images of an area outside of the fracture gap; *lower line*: the same images of an area within the fracture gap

Auregan et al. [10] detected a slight comminution in 63% of all usable closed femur fractures created according to Bonnarens et al. This finding greatly impedes correct segmentation. By using the same fracture model, Morgan et al. established a widely used segmentation method, called ‘semi-automated’ image segmentation. They define the outer boundary of the callus and periosteal surface of the original cortical bone of each two-dimensional (2D) image (Fig. 1c) [5, 11]. This procedure enables the volume-dependent parameters (e.g. bone volume/total volume (BV/TV) or bone mineral density (BMD)) to be determined. However, it appears to be rather theoretical, because two contours have to be drawn around each piece of bone to enclose it. First, in addition to the periosteal callus, the endosteal callus has to be considered. Second, depending on the cutting plane, additional separated islands of bone can occur in the 2D planes (particularly in the case of comminution). Contouring of all ROIs is thus extremely laborious, time-consuming and error-prone. In addition, if the contour of a ROI is not drawn directly adjacent to the bone surface and thus encloses certain amounts of air in the extra space around the bone surface, the volume-dependent parameters will be underestimated (Fig. 1g).

Therefore, we aimed to apply a fully automated method to identify and separate the callus, the original cortical bone and the marrow without requiring specific ROIs in a closed fracture model. We also aimed to assess the non-volume-dependent μCT parameters after 21 days for association with biomechanical parameters. To this end, we applied medication with the potential of influencing regeneration in diverse ways, namely by causing inhibition (diclofenac and prednisolone) or by having no effect (cefuroxime).

## Methods

### Animal model

Sixty-four adult male rats (CRL: WI, aged 16 weeks, mean weight ± SD 500 g ± 50 g, taken from an animal experiment) were acclimatised for 2 weeks prior to surgery. The rats were singly fed (temperature 23–25 °C, humidity 55 ± 5%, 12-h light/dark cycle) and allowed access to water and standard laboratory pellets ad libitum. The local animal research committee authorised the animal experiment in accordance with German legislative requirements (reference number of the Regierung von Oberbayern: 55.2-1-54-2531-15-08).

Anaesthesia was performed by intramuscular application of medetomidine (Medetomin, 0.15 mg/kg, Dechra Veterinary Products, ‘s-Hertogenbosch, Netherlands), midazolam (Midazolam, 2 mg/kg, Hexal AG, Germany) and fentanyl (Fentadon, 5 μg/kg, Dechra Veterinary Products, ‘s-Hertogenbosch, Netherlands). As adopted from the method first described by Bonnarens and Einhorn in 1984 [12], a Kirschner wire (K-wire; 1.0 mm) was inserted into the medullary canal of the right femur of rats in an antegrade manner followed by a closed fracture of the mid-diaphysis. Pain relief was established by subcutaneous buprenorphine injection twice a day (Buprenodale, 0.05 mg/kg, Dechra Veterinary Products). Animals were randomised and allocated to different arms (cefuroxime, diclofenac, prednisolone or control) and groups (group A: biomechanical testing; group B: μCT) (Table 1). On day 21, the rats were euthanised under isoflurane (4%) anaesthesia by an overdose of Narcoren (sodium pentobarbital 80 mg/kg BW). X-ray controls (anterior-posterior and lateral views) via c-arm (Siemens, Erlangen, Germany) were performed after intramedullary pinning, following the fracture (both intraoperatively), and post-mortem. Afterwards, bones were fresh-frozen and stored at −20 °C (biomechanics) or fixed in methanol (μCT) and stored at 4 °C. All study examiners were blinded throughout the experiments.

**Table 1:** Numbers of animals/group

Group	Cefuroxime	Diclofenac	Prednisolone	Control
A: Biomechanics	9×	8×	11×	11×
B: μCT	6×	7×	6×	6×

*Group A* biomechanics, *Group B* micro-CT

### Biomechanics

A material test machine (Wolpert TZZ 707/386, Istron Wolpert GmbH, Darmstadt, Germany) was used to apply three-point bending of the femura. The femura (*n =* 39) were placed horizontally with the anterior surface upwards. A distance of 15 mm between the bearing and loading bars for each femur was used as recommended by Turner and Burr [13].

A round-ended intender with a displacement rate of 5 mm/min was directed vertical to the mid-shaft of the femur to apply a bending load until failure (breaking load). The failure criterion was defined as a force reduction of 80%. The termination criterion was defined as a force reduction of >50 N. The test programme Test&Motion (DOLI Elektronik GmbH, München, Germany) was used to determine, from the load-displacement diagram, the highest point (breaking load (N)) and regression (stiffness (N/mm)). Both failure load and stiffness were detected for the right femura.

### Micro-CT

**Fig. 2:**
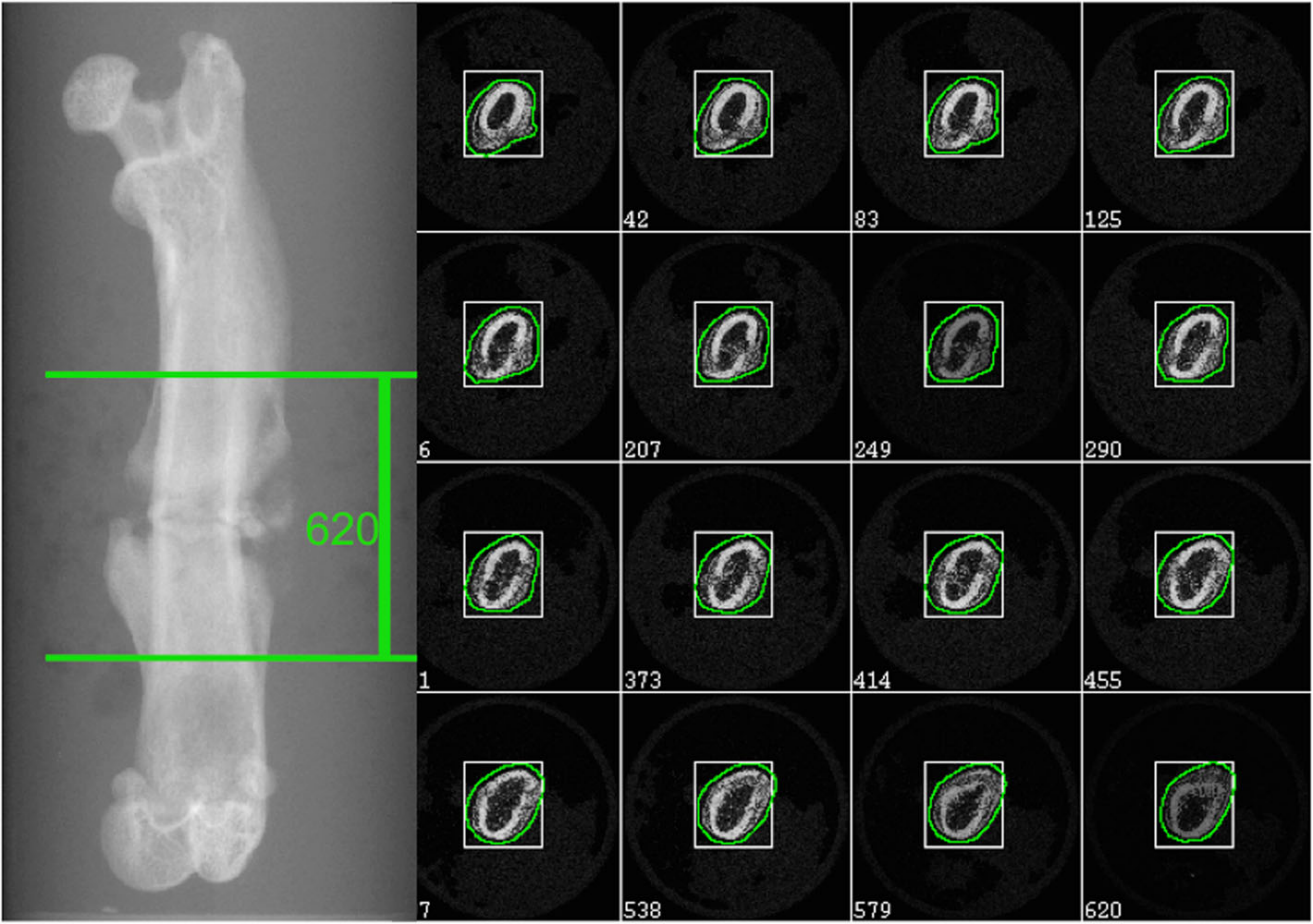
Scout view (*left*) and respective scanning area within two reference lines. Overview of 16 2D grey scale images out of the 620 slices with ROIs. The ROIs between the first and last slice were calculated by routine interpolation (*right*)

**Fig. 3:**
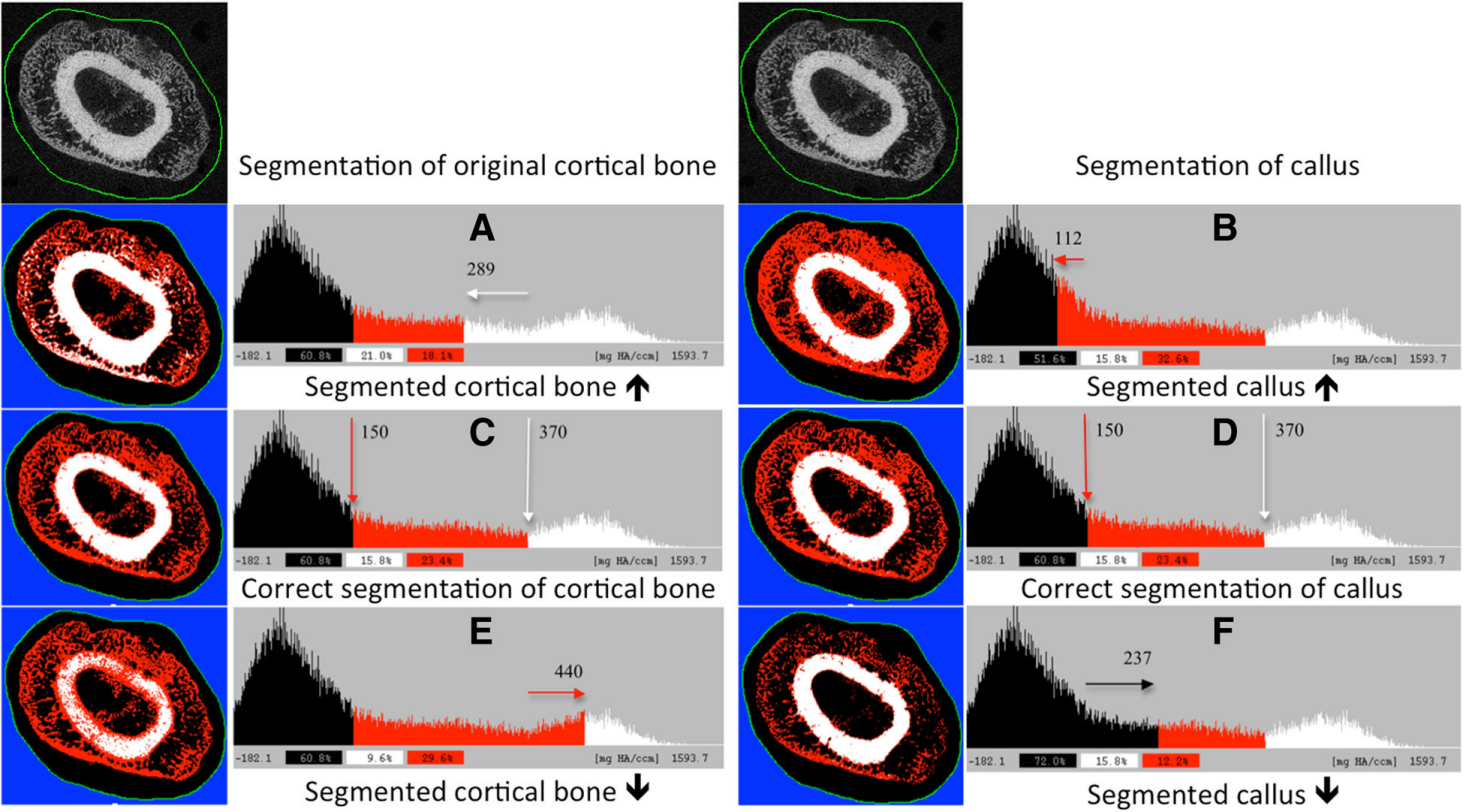
2D axial μCT (*first line*) grey value image (contour of the ROI is not intended to be drawn directly adjacent to the bone surface) of the original cortical bone, callus and air/bone marrow with corresponding fully automatically segmented images below (callus = *red*, original bone = *white*, marrow/air = *black*). Corresponding histograms (**a**–**f**) identify the three areas. In **c** and **d**, grey values higher than or equal to 150 were coloured *red*, those higher than or equal to 370 *white*, and below 150 *black*, representing correct segmentation. In the *second line*, the threshold is set to low for the original cortical bone (**a**) and callus (**b**), leading to an overestimation of the respective tissue, whereas in the *bottom line*, the threshold is set to high for the original cortical bone (**e**) and callus (**f**), leading to an underestimation of the respective tissue

The femura were scanned using an isotropic voxel size of 10 μm (55 kVp, 145 μA; μCT 40, Scanco Medical, Brüttisellen, Switzerland). The integration time was set to 200 ms. Images were reconstructed with 2048 × 2048 pixels per cross section. Before measurement, a scout view was obtained, and the scanning area of 6200 μm (slice increment 10 μm) covering both sides of the fracture gap (each 3.1 mm) was determined within two reference lines (Fig. 2). An arbitrary circle wide enough to enclose the grey values of all slices was manually drawn on slice 1 and on slice 620 and served as the ROI. Interpolation between the first and last slices was performed by the built-in software to connect the ROIs (Figs. 2 and 3 first line).

This approach did not allow for separation of the cortical bone, trabecular bone and air; therefore, thresholding was used for this purpose [14]. Global thresholding was visually determined by two independent examiners (based on slice-wise 2D comparisons between the grey scale and segmented images of all samples and histograms) and, essentially, by the associated histogram (Fig. 3a–f), in which the relative frequency of each density value (mg HA/cm^3^) or grey value (dimensionless) was displayed by the height of a corresponding line. Local maxima indicated areas representing (larger) objects or background (black). Three areas could be clearly identified in the histogram: a black area (left peak), which represents air or marrow, a red area (middle plateau), which illustrates the callus, and a white area (right maximum), which represents the original cortical bone. All pixels with grey values higher than the threshold were coloured using the same parameters for callus (sigma (0.8), support (1) and threshold (150)) and original cortical bone (sigma (1.5), support (3) and threshold (370)). Pixels with grey values less than 150 were coloured black.

**Fig. 4:**
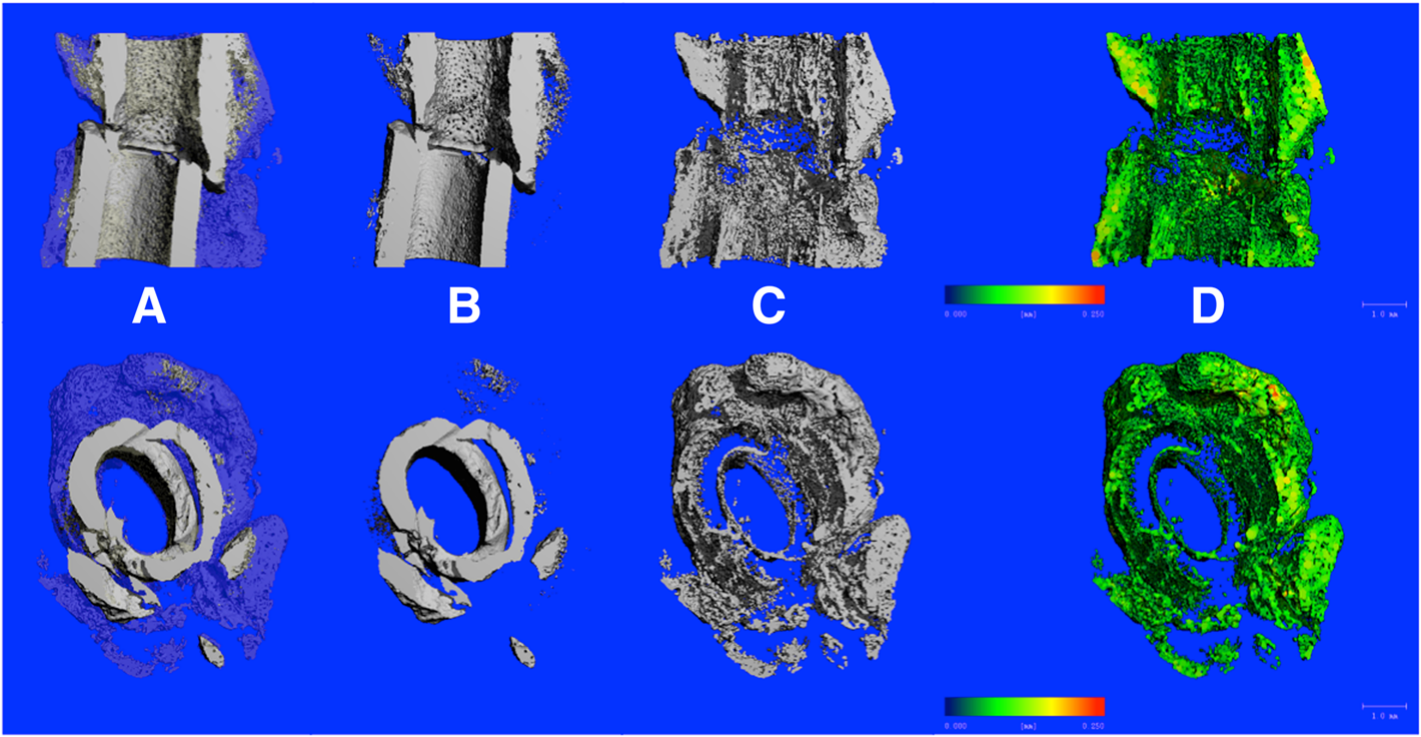
Coronal half-sliced (*first line*) and axial (*second line*) 3D μCT reconstruction (Two Thresholding Algorithm) of a multi-fragmented fracture: callus (*blue* and *semitransparent*) and cortical bone (*grey*) (**a**), isolated cortical bone (**b**) and isolated peri- and endosteal callus as grey image (**c**) and colour-coded (**d**) The coloured local thickness map/histogram illustrates the thickness distribution of the trabeculae in **d** Note the isolated fractions of trabecular-like struts thicker than 1 mm in diameter (*yellow*) and the missing outer periosteal callus of a specimen from the prednisolone group. All images represent the same specimen. *Scale bar* = 1 mm

A constrained 3D Gaussian filter partially suppressed the noise in the volumes. After reconstruction of the data, the micro-structural parameters were analysed based on the selected volume of interest (VOI) to obtain the 3D evaluation. A standard convolution-backprojection procedure with a Shepp and Logan filter was used to reconstruct 3D CT images. All image processing steps were automatically conducted by using image processing language (IPL, Institute for Biomedical Engineering, ETH and University of Zürich). The following non-volume-dependent parameters could be determined: bone volume (BV, mm^3^), tissue mineral density (TMD, mg HA/cm^3^) and bone mineral content (BMC, defined as the callus BV multiplied by TMD, mg) [5]. The structure model index (SMI, dimensionless), degree of anisotropy (DA, dimensionless), bone surface (BS, mm^3^) and trabecular thickness (Tb. Th., mm, Fig. 4) were determined as structural measures [14]. A correction algorithm was used based on a 1200-mg hydroxyapatite (HA)/cm^3^ phantom to reduce the beam-hardening effects. The density calibration data enabled the calculation of TMD and BMC. Two-voxel ‘peeling’ was used when calculating TMD to minimise partial volume effects.

### Statistical analysis

Pearson’s correlation coefficients between groups were used to examine the association of the median results of micro-CT and biomechanics (unpaired data). Additionally, a linear regression model was fitted to the data with biomechanical data as the dependent variable and μCT data as the independent variable. All statistical analyses were performed using GraphPad Prism Version 6.00 (GraphPad Prism Software® San Diego, USA). All tests were conducted on exploratory two-sided 5% significance levels.

## Results

All in all, 64 adult male rats were examined. Figure 1 displays 2D axial μCT grey value images (a and e), contoured images according to the methods described by Nyman (b and f) and Morgan (c and g) and corresponding fully automated segmented images (d and h, callus = red, original bone = white, marrow/air = black) [5, 9]. The contouring of the ROI, as applied in our study, is shown in Fig. 3; notably, the contour does not need to be drawn directly adjacent to bone surface. Conventional contouring with a single ROI adjacent to the callus (Fig. 1b, f) led to overestimation of the TV because it includes all tissue within the outer contour in the equation (e.g. bone marrow, air, cortical bone). As a consequence, the volume-dependent parameters (BV/TV and BMD) are underestimated. Two contours (outer callus = green line and outer cortical bone = red line) enclosing the callus according to conventional contouring enabled the evaluation of all parameters within the ROI in an area not corresponding to the fracture (Fig. 1c). No conventional contouring was possible around the fracture gap, especially if slight comminution was present (Fig. 1g, segmented as in c). Figure 1h shows an adequate separation of all islands of the callus and cortical bone via fully automated segmentation, even within the fracture gap.

Fully automated segmentation was performed with three different thresholds for both the cortical bone (Fig. 3a, c, e) and callus (Fig. 3b, d, f). Using incorrect thresholds for the callus or cortical bone leads to misinterpretation of the segmentation. For instance, a low threshold for cortical bone causes false-positive cortical bone segmentation at the expense of the callus. This erroneous segmentation can be observed as misplaced white (cortical) pixels in the callus (Fig. 3a). In Fig. 3c, d, correct thresholding has been applied, and the optimal distinction between the marrow, callus and cortical bone is obtained with the fully automated method.

**Fig. 5:**
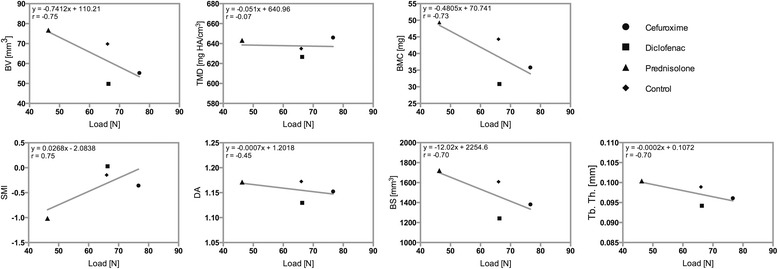
Scatterplots with regression line showing the association between biomechanics (*Load*) and μCT (*BV*, *TMD*, *BMC*, *SMI*, *DA*, *BS* and *Tb. Th.*). The estimated regression model and correlation coefficient (*r*) are also indicated

A strongly negative correlation was found between the median BV in the four groups evaluated by μCT and the median load in the four groups evaluated by biomechanics (*r =* −0.75, *p =* 0.248). The estimated regression equation was BV = −0.7412 load + 110.21. No correlation was observed between TMD and the load (*r =* −0.07, *p =* 0.926), and the estimated regression equation was TMD = −0.051 load + 640.96. A strongly negative correlation was noted between BMC and the load (*r =* −0.73, *p =* 0.269). The estimated regression equation was BMC = −0.4805 load + 70.741. SMI and the load were strongly correlated (*r =* 0.75, *p =* 0.255), whereas DA and the load were weakly, negatively correlated (*r =* −0.45, *p =* 0.551). The estimated regression equations were SMI = 0.0268 load − 2.0838 and DA = −0.0007 load + 1.2018. A strongly negative correlation was determined between BS and the load and between Tb. Th. and the load (*r =* −0.70, *p =* 0.296; *r =* −0.70, *p =* 0.300, respectively). The estimated regression equations were BS = −12.02 load + 2254.6 and Tb. Th. = −0.002 load + 0.1072 (Fig. 5, Table 2).

**Table 2:** Median and interquartile range (IQR)

	Load	Stiffness	BV	TMD	BMC	SMI	DA	BS	Tb. Th.
C	76.63	93.52	55.28	646.01	35.81	−0.3583	1.1524	1381.08	0.0961
	27.55	107.48	12.24	18.80	7.90	0.90	0.04	336.06	0.006
D	66.33	59.28	49.81	626.60	30.83	0.0291	1.1296	1241.11	0.0942
	32.72	56.53	13.95	30.91	9.97	0.72	0.06	404.17	0.007
P	46.28	60.74	76.73	643.25	49.39	−1.0147	1.1712	1720.34	0.1004
	32.81	51.51	21.69	15.70	13.55	1.42	0.06	529.21	0.007
K	66.00	107.76	69.82	634.98	44.32	−0.146	1.1725	1607.77	0.0989
	51.77	136.57	27.49	25.91	15.66	1.19	0.09	526.41	0.004

*C* cefuroxime, *D* diclofenac, *P* prednisolone, *K* control

**Fig. 6:**
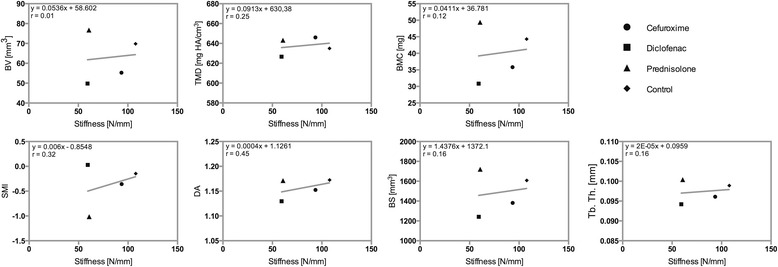
Scatterplots with regression line demonstrating the association between biomechanics (*Stiffness*) and μCT (*BV*, *TMD*, *BMC*, *SMI*, *DA*, *BS* and *Tb. Th.*). The estimated regression model and correlation coefficient (*r*) are also indicated

A weak correlation was shown between the median BV in the four groups evaluated by μCT and the median stiffness in the four groups evaluated by biomechanics (*r =* 0.10, *p =* 0.896). The estimated regression equation was BV = 0.0536 stiffness + 58.602. A weak correlation was noted between TMD and the stiffness (*r =* 0.25, *p =* 0.748), and the estimated regression equation was TMD = 0.0913 stiffness + 630.38. A weak correlation was also shown between BMC and the stiffness (*r =* 0.12, *p =* 0.881). The estimated regression equation was BMC = 0.0411 stiffness + 36.781. SMI and the stiffness were weakly correlated (*r =* 0.32, *p =* 0.682), as were DA and the stiffness (*r =* 0.45, *p =* 0.546). The estimated regression equations were SMI = 0.006 stiffness – 0.8548 and DA = 0.0004 stiffness + 1.1261. Weak correlations were also observed between BS and the stiffness and between Tb. Th. and the stiffness (*r =* 0.16, *p =* 0.840; *r =* −0.16, *p =* 0.838, respectively). The estimated regression equations were BS = 1.4376 stiffness + 1372.1 and Tb. Th. = 2E−05 stiffness + 0.0959 (Fig. 6, Table 2).

## Discussion

Slight comminution was reported for a high percentage of all usable closed femur fractures created according to Bonnarens et al. [10]. This finding extensively interferes with correct segmentation and requires reliable, easy segmentation procedures. Therefore, we applied a fully automated method to identify and separate all parts of the callus, original cortical bone and marrow without requiring determination of specific ROIs. In addition, we assessed the non-volume-dependent μCT parameters for association with biomechanics.

Contrary to our findings, the ‘semi-automated’ image segmentation method, as described by Morgan et al. in 2009 and again in 2016, precludes the measurement of the endosteal callus. However, the latter contributes to the stability of the entire structure. Morgan et al. also remark that mechanical contributions occur at the level of the ‘entire’ callus [5, 11]. Dickson et al. showed increased biomechanical stability with increased endosteal calcified tissue volume after femur fractures [15].

Our study was not originally designed to provide a systematic correlation or comparison of methods; we instead aimed to assess the influence of medication on fracture healing (data not shown). However, when interpreting these data, uncertainty was apparent concerning the impact of various parameters. Therefore, we further evaluated the association between μCT and biomechanical data between different animals. Based on the different processing of the two methods, we could not compare the same femura for association of single values between μCT and biomechanics. However, we could compare the medians of the groups, resulting in fewer values with a minor impact. Nevertheless, conclusions can be drawn from the results. The weight range of our rats was very narrow such that results for femura from different rats are comparable [13].

Unlike the reported positive correlations between BV and biomechanics of unfractured femura [16] and the very strong positive correlations between BMC and the structural strength using pQCT and DXA (*r* > 0.95) [6, 17, 18], our results yielded a strongly negative correlation between the load and BV (*r =* −0.75), BMC (*r =* −0.73) or BS (*r =* −0.70). However, the study approach for a regenerating callus is completely different from studies evaluating unfractured bone [19]. In addition, the level of fracture comminution is positively correlated with the amount of callus [10]. Nyman et al. previously showed an inverse correlation between μCT callus BV and strength (the body tries to compensate for the reduced stability) after 28 days, placing these results in a logical context [9]. With regard to TMD, no relevant association could be seen between the measures (*r =* −0.08). Shefelbine et al. reported a good predictive value of biomechanical measurements in osteotomy models (clear gap) for density measurements. However, in more complex fractures (fragmentation), which are frequently obtained by closed fracture mechanisms according to Bonnarens et al., density measurements lead to poor predictions of fracture callus biomechanics [10, 19].

Again, in contrast to our initial expectation, the SMI had a strongly positive correlation with the breaking load (*r =* 0.75). During ageing (osteoporosis) and disease, plates are perforated and connecting rods are dissolved as part of a transition from more stable (and dense) plate-like (SMI ≤0) to less stable rod-like (SMI = 3) trabeculae [2, 20]. The SMI of the prednisolone group was considerably lower (−1.30) than the SMI of the other groups. In combination with the lowest load, the latter led to the positive association between the groups. Unlike the biomechanical evidence, the trabecular structure of the prednisolone-affected callus indicates there is a more stable callus. DA, representing the orientation of the trabecular structure responsible for the anisotropic properties of the bone, revealed a weakly negative correlation (−0.45). [21]. Tb. Th. revealed a strongly negative correlation (*r =* −0.70). The notable, but unexpectedly, lowest load (46.28) compared with the highest Tb. Th. (0.1004) in the prednisolone group contributed to the negative association between the groups. Qualitative evaluation of the colour-coded μCT reconstructions, representing the Tb. Th., showed slightly thicker trabecular-like struts in the prednisolone group compared with those of the other groups (Fig. 4d). Prednisolone calluses seemed to have a lower porosity (note the increased TMD) compared with the other groups. Around the 21st day, the cartilage and original cortical bone tissue are generally remodelled, increasing the void spaces between the built trabeculae [22]. Histologically, a delayed healing of 5 days has previously been described after prednisolone medication, which might explain the thicker trabeculae and the increased thickness compared with that of the other groups [23]. Additionally, the thicker (Tb. Th.) and plate-like (SMI) trabeculae, assumed to have higher stability, do not increase the stability because of the missing outer periosteal callus fracture gap-bridging that is typically observed within this group (Fig. 4c) [22]. Consistent with our findings, increased outer bony bridging has previously been demonstrated to be able to enhance the correlation of μCT parameters with strength [9]. Therefore, bridges across the fracture gap, particularly the outer periosteal callus, can be considered relevant for biomechanical properties, even if they only slightly contribute to the total callus. Our study has shown that the localisation of the callus is important for biomechanical properties; this might explain the unexpected correlations between several biomechanical and structural measures (BV, SMI and Tb. Th.) [22, 24]. Therefore, therapies that enhance bone healing and lead to a bridging callus with increased load might lead to stronger, positive (vice versa for SMI) associations between the modalities and reach statistical significance [8]. This implication is particularly important in a clinical sense because an increasing number of studies are assessing the influence of medication (e.g. rivaroxaban and tadalafil) on fracture healing and bone remodelling, respectively [25, 26].

The breaking load is considered a more precise parameter compared with stiffness. Because of the well-known strong correlation between the methods, the latter can strengthen the results collected based on the breaking load but is not discussed here [27].

## Conclusion

The fully automated segmentation procedure for identifying and separating the callus components, the original cortical bone and the marrow represents a reliable, easy method for assessing non-volume-dependent μCT parameters. Given the increasing number of studies evaluating drugs with the potential of influencing bone regeneration, fully automated segmentation appears to be clinically important. The localisation of the bone, especially the outer bridging callus, might play a significant role in the interpretation of the data.

Soft tissue also contributes to the mechanical properties of the callus, particularly when the callus tissue transforms to cartilage before mineralisation [19]. In the future, non-invasive and non-destructive contrast-enhanced (in vivo) μCT imaging of the cartilage might provide complementary information for improving the prediction of the callus mechanical properties and thus reduce the number of animals needed in studies.
